# Prediction and Mapping of Intraprostatic Tumor Extent with Artificial Intelligence

**DOI:** 10.1016/j.euros.2023.05.018

**Published:** 2023-06-13

**Authors:** Alan Priester, Richard E. Fan, Joshua Shubert, Mirabela Rusu, Sulaiman Vesal, Wei Shao, Yash Samir Khandwala, Leonard S. Marks, Shyam Natarajan, Geoffrey A. Sonn

**Affiliations:** a Department of Urology, David Geffen School of Medicine, Los Angeles, CA, USA; b Avenda Health, Inc., Culver City, CA, USA; c Department of Urology, Stanford University School of Medicine, Stanford, CA, USA; d Department of Radiology, Stanford University School of Medicine, Stanford, CA, USA; e Department of Medicine, University of Florida, Gainesville, FL, USA

**Keywords:** Artificial intelligence, Magnetic resonance imaging, Prostatic neoplasms, Surgical margins, Surgical pathology

## Abstract

**Background:**

Magnetic resonance imaging (MRI) underestimation of prostate cancer extent complicates the definition of focal treatment margins.

**Objective:**

To validate focal treatment margins produced by an artificial intelligence (AI) model.

**Design, setting, and participants:**

Testing was conducted retrospectively in an independent dataset of 50 consecutive patients who had radical prostatectomy for intermediate-risk cancer. An AI deep learning model incorporated multimodal imaging and biopsy data to produce three-dimensional cancer estimation maps and margins. AI margins were compared with conventional MRI regions of interest (ROIs), 10-mm margins around ROIs, and hemigland margins. The AI model also furnished predictions of negative surgical margin probability, which were assessed for accuracy.

**Outcome measurements and statistical analysis:**

Comparing AI with conventional margins, sensitivity was evaluated using Wilcoxon signed-rank tests and negative margin rates using chi-square tests. Predicted versus observed negative margin probability was assessed using linear regression. Clinically significant prostate cancer (International Society of Urological Pathology grade ≥2) delineated on whole-mount histopathology served as ground truth.

**Results and limitations:**

The mean sensitivity for cancer-bearing voxels was higher for AI margins (97%) than for conventional ROIs (37%, *p* < 0.001), 10-mm ROI margins (93%, *p* = 0.24), and hemigland margins (94%, *p* < 0.001). For index lesions, AI margins were more often negative (90%) than conventional ROIs (0%, *p* < 0.001), 10-mm ROI margins (82%, *p* = 0.24), and hemigland margins (66%, *p* = 0.004). Predicted and observed negative margin probabilities were strongly correlated (R^2^ = 0.98, median error = 4%). Limitations include a validation dataset derived from a single institution's prostatectomy population.

**Conclusions:**

The AI model was accurate and effective in an independent test set. This approach could improve and standardize treatment margin definition, potentially reducing cancer recurrence rates. Furthermore, an accurate assessment of negative margin probability could facilitate informed decision-making for patients and physicians.

**Patient summary:**

Artificial intelligence was used to predict the extent of tumors in surgically removed prostate specimens. It predicted tumor margins more accurately than conventional methods.

## Take Home Message

A multimodal artificial intelligence (AI) model, trained to estimate prostate cancer probability in three dimensions, was retrospectively validated using prostatectomy data. AI-generated focal treatment margins outperformed conventional techniques. Furthermore, the AI model predicted negative margin probability accurately.

## 1 Introduction

Focal therapy (FT) is gaining acceptance as an alternative to conventional whole-gland treatment for patients with intermediate-risk prostate cancer (PCa) [1]. Leveraging a variety of ablative technologies [2], [3], [4], [5], [6], FT has the potential to preserve quality of life while conferring metastasis-free and overall survival rates comparable with radical prostatectomy [7]. Most FT studies rely upon magnetic resonance imaging (MRI) to identify and delineate PCa foci, but MRI-visible regions of interest (ROIs) consistently underestimate the true size and extent of PCa [8], [9], [10]. Thus, treatment margins beyond MRI-visible tumor boundaries are critical to the success of FT.

Margins defined using conventional approaches have commonly treated the entire tumor-bearing hemisphere [2], [11], [12], [13]. However, hemigland ablation is often suboptimal, resulting in the treatment of large volumes of benign tissue and undertreatment of bilateral tumors [14]. Others have applied a 1-cm uniform margin around ROI(s) [15], [16] despite the frequently asymmetrical nature of tumor extension [8]. Both of these strategies fail to account for patient-specific imaging, biopsy, and biomarker data that may warrant larger or smaller margins.

Artificial intelligence (AI) techniques have the potential to improve the accuracy of prostate tumor delineation [17], [18], [19], [20], [21]. Furthermore, tracked biopsy data derived from MRI-ultrasound fusion devices [22] could complement imaging data. An AI-driven approach that incorporates multimodal biopsy, imaging, and clinical features may better define treatment margins than MRI alone. In this retrospective study, we estimated the extent of clinically significant disease and the likelihood of negative surgical margins using an AI model recently cleared by the US Food and Drug Administration (“Avenda Health AI Prostate Cancer Planning Software,” K221624). We hypothesized that tumor margins defined by AI would surpass the accuracy of conventional margins, potentially improving the outcomes of focal treatment.

## 2 Patients and methods

First, the AI model was trained using tracked biopsy data. Next, the encapsulation confidence score (ECS) was developed using a whole-mount (WM) histopathology calibration dataset. Lastly, the AI model and ECS were retrospectively evaluated using a WM test dataset, which was independent from training and calibration data.

### 2.1 AI model training

**Fig. 1:**
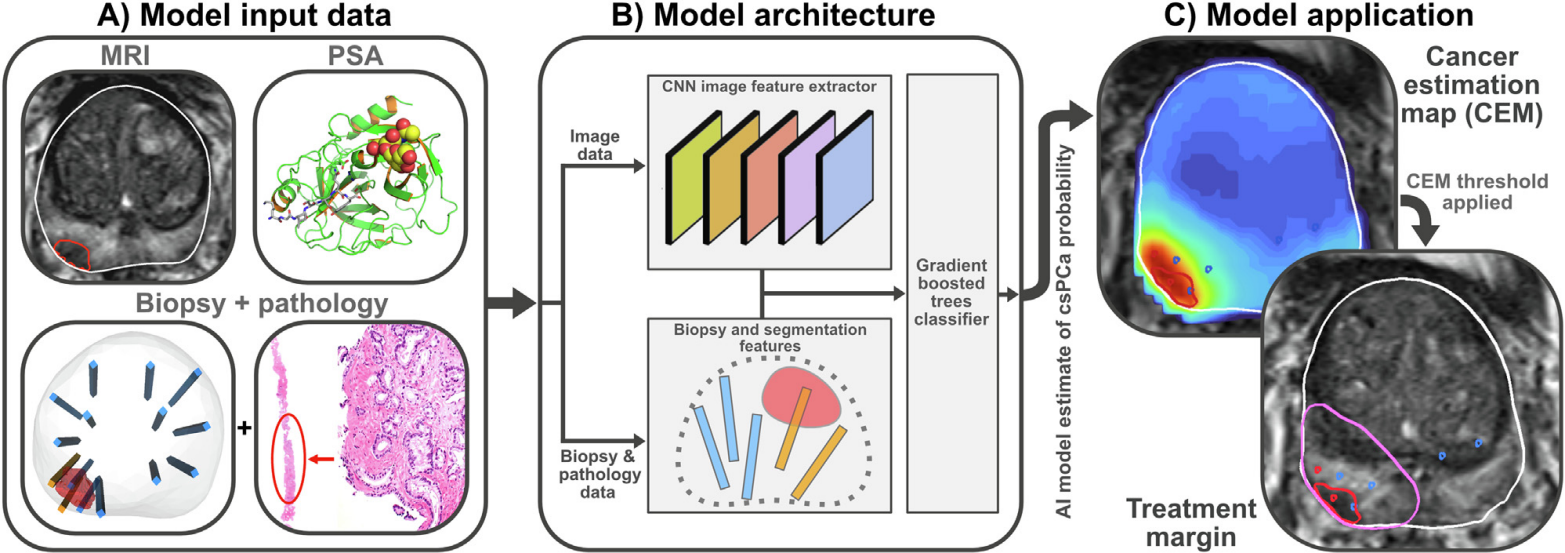
Overview of artificial intelligence (AI) model input and output, including: (A) input data, which consists of T2-weighted magnetic resonance imaging (MRI), serum prostate-specific antigen (PSA), and biopsy core locations with pathology labels; (B) high-level model architecture, wherein image features are generated via a convolutional neural network and other features are engineered from biopsy and pathology data; and (C) application of the model to produce a cancer estimation map (CEM) showing voxel-level predictions of clinically significance prostate cancer (csPCa). The CEM is thresholded in order to produce a treatment margin.

The AI model was trained using multi-institutional, consecutively accrued biopsy data. Model input data (Fig. 1A) were multimodal, consisting of T2-weighted MRI, prostate and ROI segmentations, biopsy coordinates derived from an MRI-ultrasound fusion platform, serum prostate-specific antigen (PSA), and biopsy histopathology including cancer length and International Society of Urological Pathology (ISUP) grade.

A high-level model architecture is shown in Figure 1B. For each sample point, an eight-layer three-dimensional (3D) convolutional neural network [23] generated an image feature vector from T2-weighted MRI. Additional features were generated based on serum PSA and spatial relationships between biopsy cores, ROIs, and the prostate capsule. Spatial and image features were then concatenated and classified via a gradient-boosted decision tree (XGBoost [24]). Five-fold cross validation was used to generate five models, which were averaged to produce a single prediction between 0 and 1: the estimated probability of clinically significant PCa (csPCa, defined as ISUP grade ≥2) at the sample's location.

This model was subsequently used to predict csPCa probability at all intraprostatic MRI voxels, creating a 3D cancer estimation map (CEM). The CEM was thresholded and smoothed to produce treatment margins encapsulating the regions of elevated csPCa risk (Fig. 1C). Additional details on AI model training are provided in the Supplementary material.

### 2.2 Development of the ECS

In order to provide a clinical context for FT planning, the AI model output was correlated with the probability of negative margins, that is, margins that fully encapsulated all csPCa. Whereas the AI model was trained using biopsy data, subsequent calibration and testing used WM histopathology as ground truth. The following inclusion criteria were used to select plausible FT candidates from a WM database of prostatectomy cases consecutively accrued between 2013 and 2017. The inclusion criteria were based on the preoperative data only:

1. At least one ROI, defined using the Prostate Imaging Reporting and Data System (PI-RADS) v2 [25], harbored ISUP grade 2–3 csPCa on biopsy
2. Clinically significant PCa appeared restricted to a single hemisphere or the anterior gland
3. Patient had no prior radiation or ablative treatment

Applying these inclusion criteria, 50/124 (40%) WM cases were eligible for the ECS calibration dataset. Ground truth tumor labels were defined by a pathologist, and all PCa-positive foci containing ≥5% pattern 4 or 5 disease were considered to be csPCa, including interconnected subregions dominated by pattern 3. WM slides were registered to preoperative MRI with the aid of patient-specific 3D-printed molds [8], and tumor labels were interpolated into 3D surfaces. Patient and imaging characteristics for calibration data are summarized in the Supplementary material (Supplementary Tables 2 and 4).

**Fig. 2:**
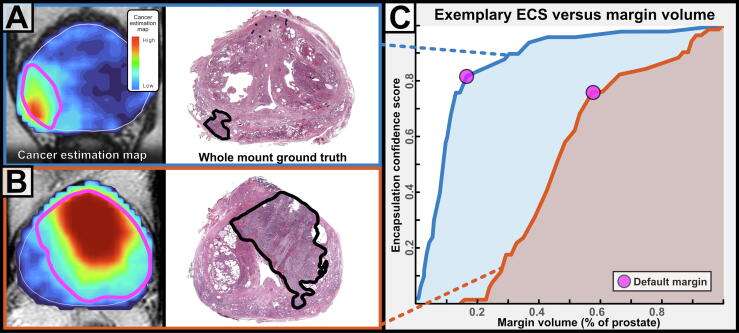
Plot of encapsulation confidence score (ECS) versus margin volume (% of the prostate) and selection of default margins for two exemplary cases. (A) A small tumor and “cool” cancer estimation map (CEM). (B) A large tumor and “hot” CEM. Cancer estimation maps are shown on the left, with the default margin outlined in pink. Corresponding whole-mount pathology images are shown in the middle, with clinically significant tumor regions highlighted in black. (C) ECS versus margin volume curves and default margin selection for the same exemplary cases.

For each case in the ECS calibration dataset, 3D margin surfaces were produced by iteratively thresholding the CEM. The proportion of cases with negative margins was recorded at each CEM threshold (Supplementary material and Supplementary Fig. 1). The resulting lookup table comprised the basis of the ECS, a novel metric intended to facilitate the selection of FT margins. When the ECS is plotted against margin volume as a percentage of the prostate (Fig. 2), physicians can prospectively assess patient-specific treatment success probability versus treatment burden.

### 2.3 AI model and ECS evaluation in an independent test set

The test dataset was wholly independent of model training data; it was drawn from a different region and institution (Stanford University), a different population, and a different MRI vendor. The same inclusion criteria listed in Section 2.2 were applied to consecutively accrued radical prostatectomy cases, resulting in a test dataset of *N* = 50 patients.

All MRI scans were acquired at 3 Tesla; other parameters varied (Supplementary material and Supplementary Table 5). T2-weighted MRI was the only image input of the AI model, although it also incorporated ROIs that were prospectively identified using multiparametric MRI (T2-weighted, diffusion-weighted, and perfusion-weighted images) according to the PI-RADS criteria. ROIs were drawn on T2-weighted MRI by a cohort of 12 radiologists with varying levels of experience (1–25 yr) during routine clinical practice.

Biopsy cores were sampled transrectally from a 12-core systematic template and one or more ROIs using an MRI-ultrasound fusion platform (Artemis; Eigen, Grass Valley, CA, USA). Biopsy detected ISUP grade 2 disease in 30 patients (60%) and grade 3 in 20 patients (40%). Prostatectomy was performed between 2014 and 2019, and ground truth was defined by registering WM histopathology annotations to preoperative MRI [26]. Descriptive statistics for the WM test dataset are presented in Table 1.

**Table 1:** Patient, region of interest, and biopsy core statistics of the test dataset

*Patient-level statistics (N* = *50 patients)*	Mean	Median	Q1	Q3
Patient age (yr)	63.4	65.5	60.0	69.0
Prostate volume (cc)	37.1	33.4	27.0	40.4
PSA (ng/ml)	7.6	6.9	5.6	9.0
PSAD ((ng/ml)/ml)	0.225	0.198	0.144	0.278

*ROI-level statistics (N* = *71 regions of interest)*				
PI-RADS v2 Score	Number	Of total (%)		
Grade 5	19	26.8		
Grade 4	40	56.3		
Grade 3	11	15.5		
Grade 2	1	1.4		

*Biopsy core-level statistics (N* = *966 biopsy cores)*				
ISUP grade	Number	Of total (%)		
Benign	608	62.9		
1	187	19.4		
2	127	13.1		
3	44	4.6		
4–5	0	0.0		

ISUP = International Society of Urological Pathology; PI-RADS = Prostate Imaging Reporting and Data System; PSA = prostate-specific antigen; PSAD = prostate-specific antigen density; ROI = region of interest.

CEMs were generated and thresholded to produce a set of AI model margins for each case. The model's receiver-operator characteristic was evaluated using the full spectrum of AI model margins. A single “default” AI margin was also produced using the patient-specific threshold that maximized ECS minus margin volume (percent of prostate), as demonstrated in Figure 2. The default margin was hypothesized to be optimal, as it balanced estimated efficacy against the requisite size of treatment. Default AI margins were compared with three conventional margin variants:

1. Prospectively defined PI-RADS v2 ROIs with biopsy-confirmed csPCa
2. A 10-mm margin around those ROIs
3. Hemigland margins that encapsulated all preoperatively evident csPCa, defined as the left, right, or anterior prostate hemisphere

AI and conventional margins were evaluated using various metrics (Supplementary material and Supplementary Fig. 2) including sensitivity and specificity of csPCa voxel classification, margin volume (% of prostate), and extent of missed csPCa. The negative margin rate was assessed as the proportion of margins that achieved (1) complete encapsulation of significant disease, defined as enclosure of all csPCa, and (2) complete encapsulation of the index lesion, defined as the largest-volume csPCa-bearing region. Tumor regions were considered to be satellites if discontinuous and at least 2 mm from the index lesion. Lastly, the accuracy of the ECS metric was assessed by comparing the ECS-predicted negative margin probability with the observed negative margin incidence at each CEM threshold.

All analyses were restricted to voxels between the most apical and basal WM slide, excluding regions where ground truth was not known. Wilcoxon signed-rank tests were used for pairwise comparisons, and chi-square tests were used to compare negative margin rates. Kolmogorov-Smirnov tests and a linear regression curve fit were used to evaluate ECS accuracy. Only trivial code was used for statistical analyses. All metrics were measured using automated scripts written in Matlab 2020b (Mathworks, Natick, MA, USA).

## 3 Results

Outcome measures are summarized in Table 2. The AI model effectively classified csPCa-bearing voxels, with a mean area under the curve of 0.911 (Supplementary material and Supplementary Fig. 3). AI default margins outperformed hemigland margins, with similar mean volume and specificity but superior sensitivity (97% vs 94%, *p* < 0.001) and a lesser extent of missed csPCa (1.6 vs 3.8 mm, *p* < 0.001). AI substantially improved upon the negative margin rate of hemigland margins for both csPCa (80% vs 56%, *p* = 0.01) and index lesions (90% vs 66%, *p* < 0.001). Positive hemigland margins resulted from midline extension of the index lesion (11/22, 50%), unencapsulated satellite csPCa foci (8/22, 36%), or both (3/22, 14%).

**Table 2:** Comparison of conventional margins with artificial intelligence default margins for the identification of clinically significant prostate cancer in the test dataset (*N* = 50)

	Original ROI	10-mm ROI expansion	Hemigland margins	Default AI margins
Sensitivity, mean (IQR)	37.4% (24.4–48.3%)	93.2% (98.8–100%)	94.1% (93.9–100%)	96.9 (99.5–100%)
Sensitivity vs a AI	*p* < 0.001 (significant)	*p* = 0.24 (insignificant)	*p* < 0.001 (significant)	–
Specificity, mean (IQR)	97.9% (97.4–99.6%)	63.4% (53.1–76%)	53.9% (51.3–55.9%)	51.2% (40.0–65.7%)
Specificity vs a AI	*p* < 0.001 (significant)	*p* < 0.001 (significant)	*p* = 0.515 (insignificant)	–
csPCa extent missed (mm), mean (IQR)	12.0 (6.5–13.1)	3.2 (0–1.7)	3.8 (0–3.8)	1.6 (0–0)
csPCa extent missed vs a AI	*p* < 0.001 (significant)	*p* = 0.001 (significant)	*p* < 0.001 (significant)	–
Margin volume % of prostate, mean (IQR)	5.0% (1.8–7.2%)	40.2% (25.5–51.7%)	49.7% (48.6–51%)	51.8% (36.2–62.7%)
Margin volume vs a AI	*p* < 0.001 (significant)	*p* < 0.001 (significant)	*p* = 0.527 (insignificant)	–
Negative margin rate (%), any csPCa	0/50 (0%)	37/50 (74%)	28/50 (56%)	40/50 (80%)
Negative margin rate vs b AI, any csPCa	*p* < 0.001 (significant)	*p* = 0.48 (insignificant)	*p* = 0.01 (significant)	–
Negative margin rate (%), index lesion	0/50 (0%)	41/50 (82%)	33/50 (66%)	45/50 (90%)
Negative margin rate vs b AI, index lesion	*p* < 0.001 (significant)	*p* = 0.24 (insignificant)	*p* = 0.004 (significant)	–

AI = artificial intelligence; csPCa = clinically significant prostate cancer; IQR = interquartile range.

a Signed-rank test.

b Chi-square test.

**Fig. 3:**
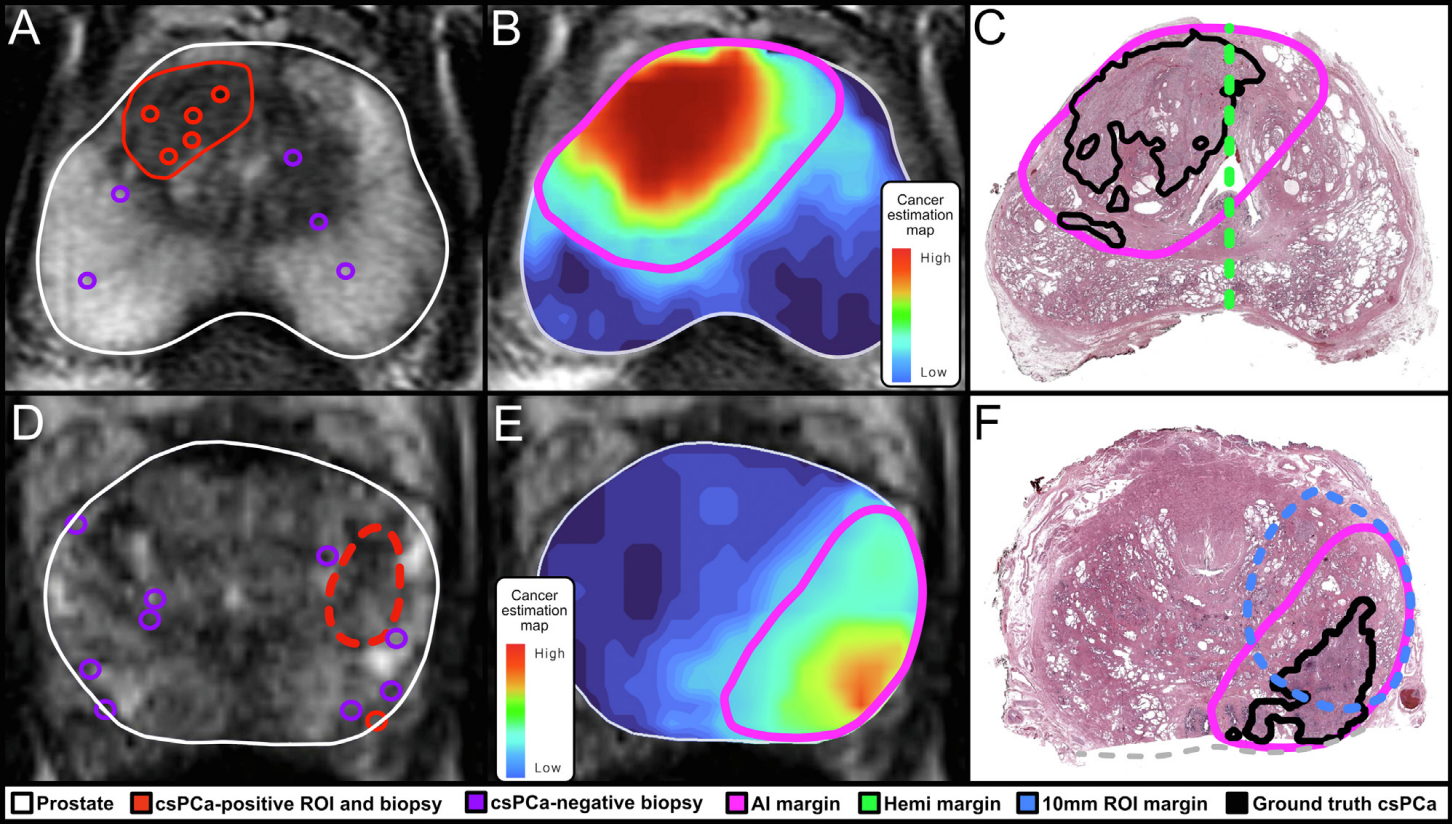
Two exemplary cases from the independent test dataset where artificial intelligence (AI) margins were negative, having succeeded in encapsulating extensions of the index lesion that were invisible on magnetic resonance imaging (MRI): (A–C) a case for which hemigland margins were positive and (D–F) a case for which 10-mm MRI region of interest (ROI) margins were positive. Figures 3A and 3D show input data including MRI, biopsy, and projected ROI location; Figures 3B and 3E show the cancer estimation map and default AI margin; and Figures 3C and 3F show whole mount histopathology along with segmentation of clinically significant prostate cancer (csPCa).

AI margins were negative for 28/28 cases (100%) with negative hemigland margins. AI margins were also negative for 12/22 cases where hemigland margins were positive, most frequently (10/12 cases) due to successful coverage of index lesion midline extensions (Fig. 3A–C). The majority of positive AI margins were due to satellite lesions (7/10 cases), most of which (6/10) were upgraded from ISUP grade 1 on biopsy to grade 2+ on WM analysis. Most of these cases had multiple contralateral cores (7/10) and/or a contralateral ROI (6/10) bearing grade 1 PCa.

Though PI-RADS ROIs with no added margin had high specificity (97.9%), mean sensitivity was very low (37.4%) and no ROI (0/50) achieved negative margins for csPCa or the index lesion. Application of 10-mm margins greatly improved ROI performance. AI margins had a lesser extent of missed csPCa (mean 1.6 vs 3.2 mm, *p* < 0.001) but also lower specificity (mean 51% vs 63%, *p* < 0.001). Sensitivity (97% vs 93%) and the negative margin rate for both csPCa (80% vs 74%) and the index lesion (90% vs 82%) were higher for AI but not significantly different. Figures 3D–F show an exemplary comparison of AI margins with 10-mm ROI margins.

**Fig. 4:**
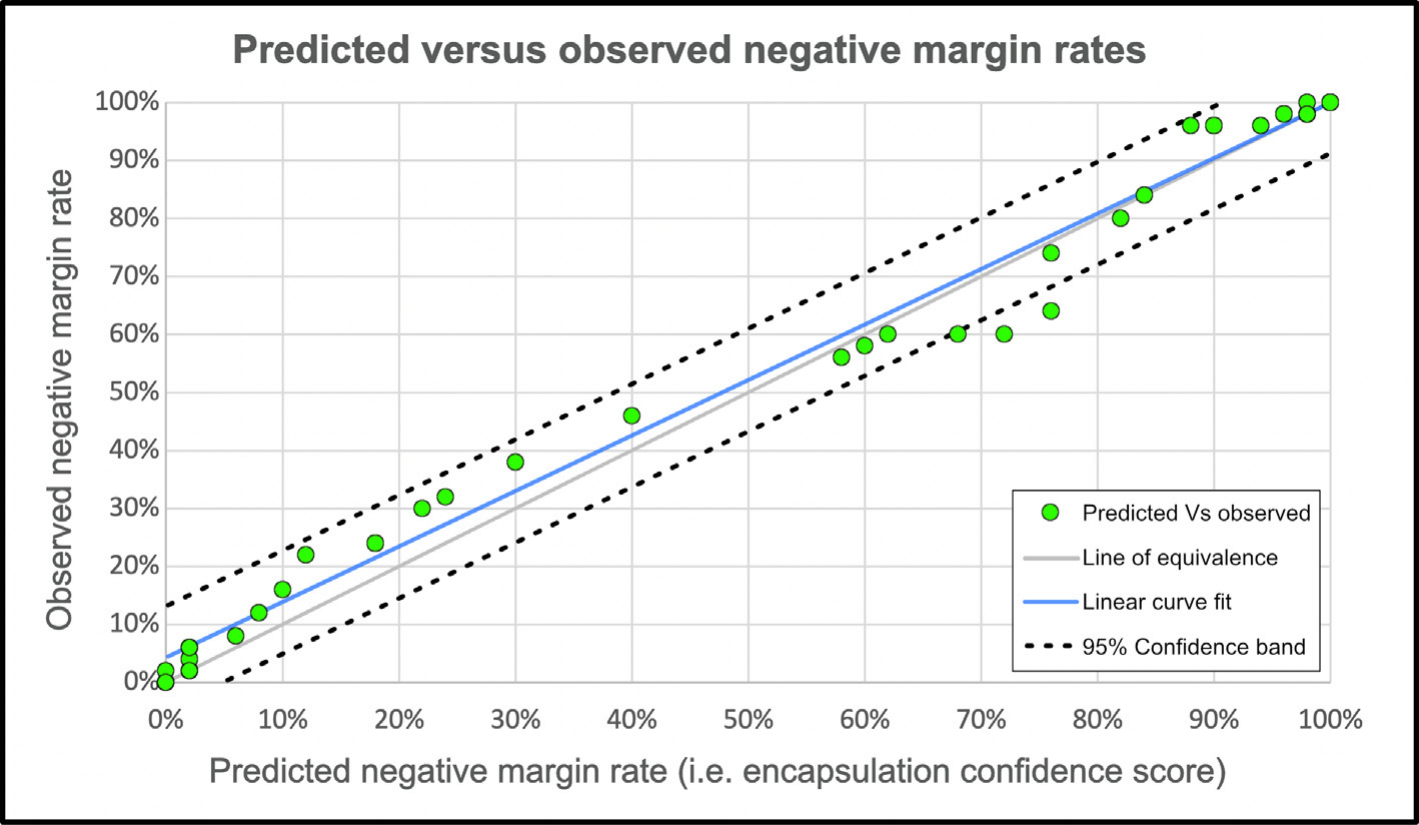
Calibration curve plotting predicted versus observed negative margin rates alongside a linear regression fit (R^2^ = 0.98). The 95% confidence bands cover a deviation of ±8% in negative margin rates.

Observed negative margin rates were strongly correlated with ECS predictions (Fig. 4), with an R^2^ value of 0.98. The median error of ECS predictions was 4% (interquartile range 2–6%), and there were no significant differences between the observed and expected negative margin rate distributions (*p* = 0.97). There were likewise no significant differences (*p* = 0.85 and *p* = 0.30) for ISUP grade 2 and 3 subpopulations, though predictions were more accurate for grade 2 (1% vs 5% median error).

## 4 Discussion

Precision management of PCa has the potential to optimize therapy while preserving quality of life, but targeted treatment first requires accurate tumor localization. PI-RADS ROIs are known to underestimate tumor extent [8], [9], [10], and in our study, treatment of the original ROI would have resulted in positive margins for every patient. It is clear that current multiparametric MRI contouring protocols, which were developed for diagnosis, are not suitable for targeted treatment. We developed a novel AI-driven approach and platform to address this shortcoming, combining multimodal information—MRI, tracked biopsy, and PSA—to produce CEMs and define optimal margins.

In the independent test set, AI margins exceeded the negative margin rate of hemigland margins for both index lesions (90% vs 66%) and any csPCa (80% vs 56%). A combination of index lesion underestimation and csPCa-bearing satellite lesions caused hemigland margins to miss csPCa in nearly half of cases. This finding is consistent with the 41–48% undetected contralateral csPCa rate reported by Johnson et al [14] and strongly suggests that, when feasible, a more patient-specific FT planning approach should be used.

AI margins decisively outperformed hemigland margins despite the nearly identical mean volume of the two approaches. However, AI was not infallible, and positive AI margins occurred most frequently (7/10) when large satellite lesions were upgraded from ISUP grade 1 on biopsy to grade 2+ on prostatectomy. The presence of MRI-visible grade 1 or multiple grade 1 biopsy cores outside a margin were risk factors for failure. Thus, due to the prevalence of multifocal csPCa and risk of positive margins, FT inclusion criteria should account for nonmicrofocal grade 1 disease foci.

The relatively strong performance of 10-mm ROI margins is consistent with the findings of Brisbane et al [22], who reported that 90% of csPCa-bearing cores were within 10 mm of an ROI. The present study was not powered for comparisons between AI and 10-mm margins; although AI margins had numerically superior sensitivity (97% vs 93%, *p* = 0.24) and index tumor negative margin rate (90% vs 82%, *p* = 0.24), these differences fell short of statistical significance. Nevertheless, a significant difference was observed in tumor extension beyond margin boundaries (mean 3.2 mm for 10-mm ROI margins vs 1.6 mm for AI margins, *p* = 0.001). This finding is consistent with the mean 13.5 mm of ROI tumor extent underestimation reported in prior publications [8].

The ECS, which predicts negative margin probability, was shown to be accurate across the full breadth of CEM thresholds. The correlation between predicted and observed negative margin rates was remarkably linear (R^2^ = 0.98), demonstrating accuracy in a test population independent of the training data. Such a tool could be of considerable clinical value, helping inform risk assessment and therapy selection. Furthermore, simple transformation of this metric (1 – ECS) yields the probability of significant tumor *outside* a margin, that is, the expected rate of post-treatment residual disease. The ECS may thus allow physicians to more precisely balance the risk of residual tumor with the risk to quality of life for an individual patient.

The CEM and ECS were developed primarily to facilitate the selection of focal treatment margins, but these may have additional applications in PCa care. For example, a randomized controlled trial of external beam radiation recently showed that biochemical disease-free survival was higher when boosting dosage to the MRI-visible index lesion [27], and CEM-defined margins have the potential to further improve “focal boosting” performance. The CEM and ECS could also be useful when planning radical prostatectomy, since neurovascular bundles outside the AI margin could be spared during surgery. There may even be applications in active surveillance programs where the CEM could aid in sampling the MRI-invisible tumor “penumbra” [22], identifying patients at risk for progression and determining therapy options for patients who desire definitive treatment.

Our study had four noteworthy limitations. First, the test population was by necessity derived from a radical prostatectomy dataset, likely with larger and more advanced disease than the average FT patient. This factor was mitigated by selecting only plausible FT candidates via the study inclusion criteria. Second, the test set was derived from a single institution, which may not be representative of broader populations. However, the algorithm was trained on multi-institutional data, wholly independent from the test dataset. Third, analyses were limited to a comparison of default AI margins against the most commonly cited conventional planning methods. The performance of margins generated by actual physician readers, with and without access to the AI model, will be investigated in a separate study. Fourth, the presented algorithm incorporates only T2-weighted imaging due to limited availability of multiparametric MRI for training and test datasets. The absence of diffusion-weighted imaging is a significant limitation since it correlates strongly with tumor presence [28] and is used to delineate peripheral zone lesions under PI-RADS guidelines [25]. Future work will include incorporation of diffusion-weighted MRI, likely bolstering AI model performance relative to static methodology such as hemigland and ROI-based margins. We also plan to evaluate AI performance in additional populations, improving statistical power and yielding more definitive comparisons.

## 5 Conclusions

An AI model was developed to define margins and assess positive margin risk during FT. In a retrospective evaluation of WM prostatectomy data, the model was shown to be accurate and effective for both of these applications. This approach could help improve and standardize focal treatment margins, potentially reducing cancer recurrence rates. Furthermore, the ECS's accurate assessment of negative margin likelihood and residual tumor risk could help facilitate informed decision-making for both patients and physicians. Prospective studies are warranted, as AI-enabled cancer mapping shows considerable promise for patient-specific treatment planning and personalized medicine.

  ***Author contributions*:** Richard E. Fan and Geoffrey A. Sonn had full access to all the data in the study and take responsibility for the integrity of the data and the accuracy of the data analysis.

  *Study concept and design*: Priester, Fan, Shubert, Natarajan, Sonn.

*Acquisition of data*: Priester, Fan, Shubert, Rusu, Vesal, Shao, Khandwala.

*Analysis and interpretation of data*: Priester, Fan, Shubert, Rusu, Vesal, Shao, Khandwala.

*Drafting of the manuscript*: Priester, Natarajan, Sonn.

*Critical revision of the manuscript for important intellectual content*: Priester, Fan, Marks, Natarajan, Sonn.

*Statistical analysis*: Priester.

*Obtaining funding*: Marks, Priester, Natarajan, Sonn.

*Administrative, technical, or material support*: Priester, Fan, Shubert, Rusu, Vesal, Shao, Khandwala, Marks, Natarajan, Sonn.

*Supervision*: Fan, Marks, Natarajan, Sonn.

*Other*: None.

  ***Financial disclosures:*** Alan Priester certifies that all conflicts of interest, including specific financial interests and relationships and affiliations relevant to the subject matter or materials discussed in the manuscript (eg, employment/affiliation, grants or funding, consultancies, honoraria, stock ownership or options, expert testimony, royalties, or patents filed, received, or pending), are the following: Alan Priester and Joshua Shubert are employees of Avenda Health. Leonard S. Marks and Shyam Natarajan are cofounders of Avenda Health. No other authors have any conflicts of interest to declare.

  ***Funding/Support and role of the sponsor***: This work was funded in part by the National Cancer Institute at the National Institutes of Health (grant R01CA218547). Avenda Health provided AI software and also played a role in the design and conduct of the study, analysis of the data and preparation of the manuscript.

  ***Data sharing***: Data are available for bona fide researchers who request it from the authors. Authors Richard E. Fan and Geoffrey A. Sonn, who are independent of any commercial entity, had full access to all the data in the study and take responsibility for the integrity of the data and the accuracy of the data analysis.

  ***Ethics statement*:** This study was retrospectively performed on extant data under the auspices of an institutional review board.

  ***Acknowledgments*:** The authors acknowledge Avenda Health for manpower and software provision.
