# Supplementary material for: Prediction and Mapping of Intraprostatic Tumor Extent with Artificial Intelligence
Source: Eur Urol Open Sci. 2023 Jun 13;54:20–7. doi: 10.1016/j.euros.2023.05.018 (PMC10403686; doi:10.1016/j.euros.2023.05.018)
Supplement: Supplementary data 1 [file mmc1.docx]

Supplementary Material

**Supplementary Methods**

MRI, targeted biopsy, and other clinical data was used to generate an AI model, the output of which was a probabilistic prediction of cancer presence at each voxel of the prostate. AI inferences were made by utilizing an ensemble network of five cancer prediction models, each consisting of an image feature extractor and a geometric feature generator. Model development is described below.

*Supplementary Methods 1.1: Description and Selection of Training Data*

The AI model was trained using multi-institutional biopsy data, consecutively accrued between 2011 and 2019. Patient-level, visit-level, ROI-level, and biopsy core-level data used for model training is summarized in Supplementary Table 1. The majority of the training data was derived from a publicly available data collection, The Cancer Imaging Archive (TCIA), entitled “*Prostate MRI and Ultrasound With Pathology and Coordinates of Tracked Biopsy (Prostate-MRI-US-Biopsy)”* (DOI: [10.7937/TCIA.2020.A61IOC1A](https://doi.org/10.7937/TCIA.2020.A61IOC1A)) [1-3]. The dataset consists of 24,783 biopsy cores derived from 1,761 biopsy procedures and 1,150 patients during tracked prostate biopsy. Additional details on the TCIA dataset are publicly available on their website, accessible through the preceding link. The TCIA data was supplemented by similarly structured imaging and biopsy data furnished from a separate institution, consisting of 8,651 biopsy cores derived from 785 biopsy procedures and 737 patients.

All ROIs were identified using multiparametric MRI (T2-weighted, diffusion-weighted, and perfusion-weighted images) and drawn on T2-weighted MRI by radiologists with ≥10 years of experience during routine clinical practice. MRI was interpreted in adherence with the PI-RADS v2 protocol, or else using a comparable Likert-like scoring system for cases predating PI-RADS v2. All biopsy data was acquired transrectally, using an MRI-ultrasound registration and tracking platform (Artemis, Eigen, Grass Valley CA), by urologists with ≥10 years of clinical experience. During biopsy ROIs were overlaid on ultrasound using a semi-automated, nonrigid, surface-based registration algorithm [4]. Core locations were tracked kinematically using encoders within a mechatronic arm [5]. Prior studies have reported approximately 3 mm of registration/tracking error associated with this technology [4,6-8].

Prior to training, the dataset was filtered to only include cases which possessed the requisite algorithm input data and which were potentially eligible for targeted treatment. The following inclusion criteria were applied.

1. Patient had ≥1 PCa-positive biopsy core.

2. Biopsy core coordinates were available and registered to MRI

3. The patient was *not* represented in the whole mount calibration or test dataset

4. No errors indicative of faulty segmentation or registration were detected

5. Patient did *not* have prior radiation or ablative treatment

6. Patient did *not* have primarily pattern 5 PCa, e.g. Gleason Score 5+5 or 5+4.

Application of the above inclusion criteria resulted in a winnowed dataset of 875 patients. The majority of these biopsy cases entailed acquisition of tissue from a 12-core systematic template and from one or more MRI region(s) of interest. The median number of cores collected per case was 15 (IQR 13-17). After removal of cores with missing or erroneous data (criteria 2 and 5 above), a median of 12 cores per case were usable for model training (IQR 9-14, 12895 cores total).

*Supplementary Methods 1.2: Image Feature Generation*

Image data consisted of T2-weighted MRI volumes; acquisition parameters are summarized in Supplementary Table 3. Since MR image resolution varied according to the device and imaging protocol, all image sets were isotropically resampled via bilinear interpolation to a voxel size of 0.5 mm^3^. The voxel intensity distribution of each MR image volume was standardized [9] in a window bounding the prostate capsule in order to compensate for interpatient and inter-scanner variability. Then, for each biopsy core in the dataset, a 16x16x48 voxel (8x8x24mm) image volume prism was aligned with the core’s trajectory and cropped around it. This window enclosed biopsy coordinates with a reasonable margin to counteract the approximately 3 mm of registration error associated with fusion biopsy [4,6-8].

An image feature extractor was applied to each of the image volume prisms. It consisted of an 8-layer 3D convolutional neural network based off the C3D architecture [10]. Each of the 8 layers consisted of a 3D convolution followed by a batch normalization layer, rectified linear unit, and max pooling layer. The output of the 8 convolutional layers was mapped to a fully connected layer, the last of which constituted an image feature vector, i.e., a concatenated list of all image features. Additionally, simple image features such as mean and maximum voxel intensity were computed and added to the image feature vector.

*Supplementary Methods 1.3: Geometric Feature Engineering*

To supplement the image-derived data, geometric features were generated for each biopsy core to represent information not necessarily present in the medical images, such as the relative positions of the prostate capsule, ROI, and other biopsy cores. All distances were computed automatically from the imported biopsy core, ROI, and prostate capsule coordinates. Engineered features included:

• ROI-Derived Features, such as distance to the nearest ROI surface

• Biopsy-derived features, such as distance to the nearest csPCa-bearing biopsy core

• Location-based features, such as distance from the prostate centroid

• Serum PSA-based features

The geometric features were ultimately aggregated into a single geometric feature vector, i.e., a concatenated list of all geometric features. Handling of missing data was feature-dependent. For example, if distance measures could not be computed due to the absence of a ROI or biopsy core subtype, the distance was set to infinity; if tissue length metadata was missing, tissue length was assumed to be equal to the biopsy needle throw length (18 mm).

*Supplementary Methods 1.4: Cancer Prediction Model Training*

The AI model was trained to predict the presence of csPCa at the location of each biopsy core. The ground-truth reference standard was the "csPCa" or "not csPCa" label for each core derived from pathology reports. All pre-processing and training steps were performed using Python version 3.7; referenced libraries included PyTorch version 1.7, SimpleITK version 1.2.4, and XGBoost version 1.0.2. Minimal data augmentation was used, consisting of left/right flipping and noise injection for MR input images.

The training data was randomly split into five subsets for five-fold cross-validation. Data was partitioned at the patient level, with samples derived from the same patient grouped together (i.e., never divided between training and validation sets) in order to minimize bias. Training took place in four stages:

1. Image feature vectors were extracted as described in Supplementary Methods section 1.2.

2. Geometric feature vectors were extracted as described in Supplementary Methods section 1.3.

3. Image and geometric feature vectors were concatenated. A gradient boosted tree ensemble classifier (XGBoost) was then trained to predict the probability of clinically significant prostate cancer (csPCa) from the input feature vector. Each of the XGBoost models had 146 decision trees, and each of the trees in the XGBoost model had a maximum depth of 7. The output was a number between 0 and 1 representing estimated probability of csPCa at that sample point.

4. Each of the 5 splits resulted in a unique cancer prediction model, all of which were combined into an ensemble network of five cancer prediction models. The cancer probability estimates for each cancer prediction model were then averaged to produce a final cancer probability estimate.

The mean accuracy and AUC of the five models on the held-out split of cross validation data was 96% ± 0.4% and 0.96 ± 0.005, respectively.

*Supplementary Methods 1.5: Margin Generation*

In prospective use, the AI model was used to infer csPCa probability in each voxel of a 3D image, resulting in a cancer estimation map (CEM). The CEM was thresholded to produce a 3D binary mask, which was then isotropically dilated by 4 mm in order to compensate for the registration/segmentation error associated with MRI-ultrasound fusion biopsy [4,6-8]. The marching cubes algorithm [11] was applied to the 3D mask to generate a surface mesh, and small disjoint manifolds (3 cc or less) were culled. Lastly, the surface was smoothed by applying a windowed sinc function interpolation kernel [12] and downsampled 70% using a quadric decimation filter [13].

**Supplementary References**

[1] Clark K, Vendt B, Smith K, et al. The Cancer Imaging Archive (TCIA): maintaining and operating a public information repository. J Digit Imaging. 2013;26(6):1045-1057.

[2] Sonn GA, Natarajan S, Margolis DJA, et al. Targeted biopsy in the detection of prostate cancer using an office based magnetic resonance ultrasound fusion device. J Urol. 2013;189(1):86-91. doi:10.1016/j.juro.2012.08.095

[3] Natarajan S, Priester A, Margolis D, Huang J, Marks L. Prostate MRI and Ultrasound With Pathology and Coordinates of Tracked Biopsy (Prostate-MRI-US-Biopsy). The Cancer Imaging Archive. https://doi.org/10.7937/TCIA.2020.A61IOC1A. Published 2020.

[4] Narayanan R, Kurhanewicz J, Shinohara K, Crawford ED, Simoneau A, Suri JS. MRI-ultrasound registration for targeted prostate biopsy. Ultrasound. 2009:991-994.

[5] Bax J, Cool D, Gardi L, et al. Mechanically assisted 3D ultrasound guided prostate biopsy system. Med Phys. 2008;35(12):5397-5410. doi:10.1118/1.3002415

[6] Karnik V, Fenster A, Bax J, et al. Assessment of registration accuracy in three-dimensional transrectal ultrasound images of prostates. Med Imaging 2010 Vis Image-Guided Proced Model. 2010;7625(February 2010):762516. doi:10.1117/12.844332

[7] Haber L, Priester A, Nassiri N, Marks L, Natarajan S. Prostate Biopsy-Site Tracking: Effect of Needle Deflection and Segmentation Errors. In: Engineering in Urology Society. ; 2016.

[8] Natarajan S. Magnetic Resonance-Ultrasound Fusion of the Prostate: Imaging for Cancer Diagnosis. 2012.

[9] Nyúl LG, Udupa JK, Zhang X. New variants of a method of MRI scale standardization. IEEE Trans Med Imaging. 2000;19(2):143-150.

[10] Tran D, Bourdev L, Fergus R, Torresani L, Paluri M. Learning spatiotemporal features with 3d convolutional networks. In: Proceedings of the IEEE International Conference on Computer Vision. ; 2015:4489-4497.

[11] Lorensen WE, Cline HE. Marching cubes: A high resolution 3D surface construction algorithm. ACM siggraph Comput Graph. 1987;21(4):163-169.

[12] Taubin G, Zhang T, Golub G. Optimal surface smoothing as filter design. In: Computer Vision—ECCV’96: 4th European Conference on Computer Vision Cambridge, UK, April 15–18, 1996 Proceedings, Volume I 4. Springer; 1996:283-292.

[13] Garland M, Heckbert PS. Surface simplification using quadric error metrics. In: Proceedings of the 24th Annual Conference on Computer Graphics and Interactive Techniques. ; 1997:209-216.

**Supplementary Tables**

Supplementary Table 1: Patient, region of interest, and biopsy core statistics of the AI training dataset

| ***Institution of Origin (N=875 Patients)*** | | |  |  |  |
| --- | --- | --- | --- | --- | --- |
|  | TCIA* | 590 | 67.4% | — |  |
|  | Other | 285 | 32.6% | — |  |
|  |  |  |  |  |  |
| ***Visit-Level Statistics (N=1145 Biopsy Visits)*** | | | |  |  |
|  |  | Mean | Median | Q1 | Q3 |
| Patient Age (Years) | | 66.3 | 66.0 | 61.0 | 72.0 |
| Prostate Volume (cc) | | 47.7 | 40.4 | 31.4 | 58.0 |
| PSA (ng/mL) | | 8.3 | 6.5 | 4.6 | 9.9 |
| PSAD ((ng/mL)/mL) | | 0.198 | 0.148 | 0.098 | 0.235 |
|  |  |  |  |  |  |
| ***ROI-Level Statistics (N=1555 Regions of Interest)*** | | | |  |  |
| PIRADS v2 Score | | Number | Of Total (%) | Of Known (%) | |
|  | Grade 5 | 304 | 19.5% | 21.0% |  |
|  | Grade 4 | 537 | 34.5% | 37.1% |  |
|  | Grade 3 | 539 | 34.7% | 37.2% |  |
|  | Grade 2 | 67 | 4.3% | 4.6% |  |
|  | Unscored | 108 | 6.9% | — |  |
|  |  |  |  |  |  |
| ***Biopsy Core-Level Statistics (N=12895 Biopsy Cores)*** | | | |  |  |
| ISUP Grade | | Number | Of Total (%) |  |  |
|  | Benign | 8383 | 65.0% |  |  |
|  | 1 | 2458 | 19.1% |  |  |
|  | 2 | 1385 | 10.7% |  |  |
|  | 3 | 390 | 3.0% |  |  |
|  | 4 | 181 | 1.4% |  |  |
|  | 5 | 98 | 0.8% |  |  |

*DOI: 10.7937/TCIA.2020.A61IOC1A

Supplementary Table 2: Patient, region of interest, and pathology statistics of the ECS calibration dataset

| ***Patient-Level Statistics (N=50 Patients)*** | | |  |  |  |
| --- | --- | --- | --- | --- | --- |
|  |  | Mean | Median | Q1 | Q3 |
| Patient Age (Years) | | 62.7 | 63.5 | 56.5 | 67.0 |
| Prostate Volume (cc) | | 36.9 | 33.7 | 28.8 | 43.1 |
| PSA (ng/mL) | | 6.8 | 6.1 | 4.7 | 7.6 |
| PSAD ((ng/mL)/mL) | | 0.199 | 0.165 | 0.125 | 0.213 |
|  |  |  |  |  |  |
| ***ROI-Level Statistics (N=70 Regions of Interest)*** | | | |  |  |
| PIRADS v2 Score | | Number | Of Total (%) |  |  |
|  | Grade 5 | 18 | 26% |  |  |
|  | Grade 4 | 28 | 40% |  |  |
|  | Grade 3 | 22 | 31% |  |  |
|  | Grade 2 | 2 | 3% |  |  |
|  |  |  |  |  |  |
| ***Tumor-Level Statistics (N=107 Tumor Foci)*** | | | |  |  |
| ISUP Grade | | Number | Of Total (%) |  |  |
|  | 1 | 51 | 47.7% |  |  |
|  | 2 | 44 | 41.1% |  |  |
|  | 3 | 12 | 11.2% |  |  |
|  | 4-5 |  | 0.0% |  |  |

Supplementary Table 3: MRI acquisition characteristics for the AI training dataset (N = 1006)

| **MANUFACTURER** | ***SIEMENS*** | ***GE MEDICAL*** | ***PHILIPS*** | ***OTHER*** |
| --- | --- | --- | --- | --- |
|  | 944 (94%) | 51 (5%) | 8 (1%) | 3 (0.3%) |
|  |  |  |  |  |
| **MAGNET MODEL** | ***SKYRA*** | ***MAGNETOM*** | ***PRISMA*** | ***OTHER*** |
|  | 760 (76%) | 53 (5%) | 42 (4%) | 151 (15%) |
|  |  |  |  |  |
| **IN-PLANE RESOLUTION** | ***0.2 - 0.4 mm*** | ***0.4 - 0.6 mm*** | ***0.6 - 0.9 mm*** |  |
|  | 101 (10%) | 247 (25%) | 658 (65%) |  |
|  |  |  |  |  |
| **THROUGH-PLANE RESOLUTION** | ***1.5 mm*** | ***3 mm*** | ***OTHER*** |  |
|  | 653 (65%) | 335 (33%) | 18 (2%) |  |
|  |  |  |  |  |
| **ECHO TIME** | ***80-125 ms*** | ***125-150 ms*** | ***175 - 225 ms*** | ***OTHER*** |
|  | 95 (9%) | 254 (25%) | 653 (65%) | 4 (0.4%) |
|  |  |  |  |  |
| **REPETITION TIME** | ***1-3 s*** | ***3-5 s*** | ***5-7 s*** | ***OTHER*** |
|  | 669 (66.5%) | 141 (14%) | 166 (16.5%) | 30 (3%) |
|  |  |  |  |  |
| **IMAGE SIZE** | ***150 - 170 mm*** | ***170 - 190 mm*** | ***190 - 225 mm*** | ***> 225 mm*** |
|  | 285 (28%) | 668 (66%) | 47 (5%) | 6 (1%) |
|  |  |  |  |  |
| **FIELD STRENGTH** | ***1.5T*** | ***3T*** |  |  |
|  | 47 (5%) | 959 (95%) |  |  |

Supplementary Table 4: MRI acquisition characteristics for the ECS calibration dataset (N = 50)

| **MANUFACTURER** | ***SIEMENS*** |  |  |  |
| --- | --- | --- | --- | --- |
|  | 50 (100%) |  |  |  |
|  |  |  |  |  |
| **MAGNET MODEL** | ***SKYRA*** | ***TRIO*** | ***VERIO*** | ***OTHER*** |
|  | 35 (70%) | 5 (10%) | 5 (10%) | 5 (10%) |
|  |  |  |  |  |
| **IN-PLANE RESOLUTION** | ***0.66 mm*** |  |  |  |
|  | 50 (100%) |  |  |  |
|  |  |  |  |  |
| **THROUGH-PLANE RESOLUTION** | ***1.5 mm*** |  |  |  |
|  | 50 (100%) |  |  |  |
|  |  |  |  |  |
| **ECHO TIME** | ***202 ms*** |  |  |  |
|  | 50 (100%) |  |  |  |
|  |  |  |  |  |
| **REPETITION TIME** | ***2200*** | ***OTHER*** |  |  |
|  | 48 (96%) | 2 (4%) |  |  |
|  |  |  |  |  |
| **IMAGE SIZE** | ***50 mm*** |  |  |  |
|  | 50 (100%) |  |  |  |
|  |  |  |  |  |
| **FIELD STRENGTH** | ***1.5T*** | ***3T*** |  |  |
|  | 2 (4%) | 48 (96%) |  |  |

Supplementary Table 5: MRI acquisition characteristics for the independent test dataset (N = 50)

| **MANUFACTURER** | ***GE MEDICAL*** | ***OTHER*** |  |  |
| --- | --- | --- | --- | --- |
|  | 46 (92%) | 4 (8%) |  |  |
|  |  |  |  |  |
| **IN-PLANE RESOLUTION** | ***0.39 mm*** | ***0.43 - 0.47 mm*** | ***0.72 - 0.94 mm*** |  |
|  | 36 (72%) | 10 (20%) | 4 (8%) |  |
|  |  |  |  |  |
| **THROUGH-PLANE RESOLUTION** | ***1.5 - 1.6 mm*** | ***3 mm*** | ***3.6 mm*** | ***4.0 - 4.2 mm*** |
|  | 4 (8%) | 16 (32%) | 9 (18%) | 21 (42%) |
|  |  |  |  |  |
| **ECHO TIME** | ***90-120 ms*** | ***120-130 ms*** | ***130-150 ms*** |  |
|  | 9 (18%) | 36 (72%) | 5 (10%) |  |
|  |  |  |  |  |
| **REPETITION TIME** | ***2-4 s*** | ***4-5 s*** | ***5-9 s*** |  |
|  | 12 (24%) | 23 (46%) | 15 (30%) |  |
|  |  |  |  |  |
| **IMAGE SIZE** | ***<200 mm*** | ***200 mm*** | ***200-400 mm*** |  |
|  | 1 (2%) | 38 (76%) | 11 (22%) |  |

**Supplementary Figures**

*
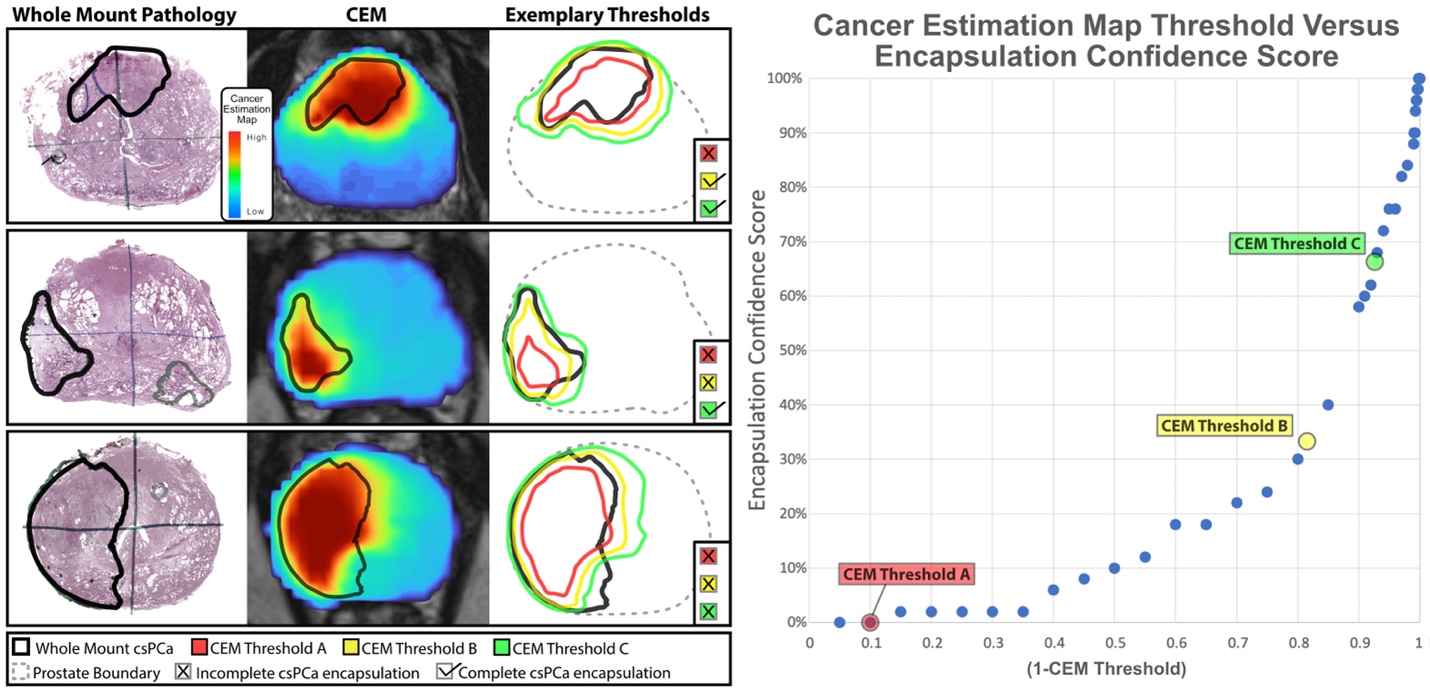
*

*Supplementary Figure 1: Formulation of the Encapsulation Confidence Score (ECS), which estimates negative margin probability of a cancer estimation map (CEM) threshold. The lefthand panel shows exemplary whole mount (WM) pathology slides and corresponding CEM images. In order to compute the ECS lookup table, CEMs are iteratively thresholded and the resulting margins are cross referenced with WM pathology. For example, in the topmost example case, the clinically significant cancer (csPCa)-bearing region contains red and orange voxels. A red CEM threshold would therefore have positive margins, but a yellow or green CEM threshold would have negative margins. Considering the three example cases in tandem, negative margins were achieved in 0/3 = 0% cases for a red CEM threshold, 1/3 = 33% for a yellow CEM threshold, and 2/3 = 66% for a green CEM threshold. The righthand panel illustrates use of these measures to define an ECS curve; the 0%, 33%, and 66% negative margin rates for the example cases are shown in red, yellow, and green. The blue dots represent the actual encapsulation confidence score curve as defined by the full N = 50 ECS calibration dataset.*

*
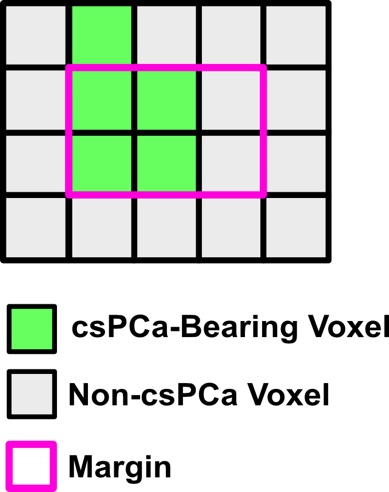
*

*Supplementary Figure 2: Illustration of outcome measures relative to clinically significant prostate cancer (csPCa)-bearing voxels. Sensitivity was defined as (number of csPCa-bearing voxels within a margin)/(total number of csPCa-bearing voxels), i.e. the volumetric percentage of tumor successfully included within margin boundaries. In this example, sensitivity = (4/5) = 0.80. Specificity was defined as (number of non-csPCa voxels outside a margin)/(total number of non-csPCa voxels), i.e. the volumetric percentage of benign tissue excluded from the margin and thus spared from unnecessary treatment in the context of FT. In this example, specificity = (13/15) = 0.87. The extent of missed csPCa was defined as the largest distance between a margin surface and unencapsulated csPCa; if all csPCa was encapsulated, its value was zero. In this example, missed csPCa extent = 1 voxel.*


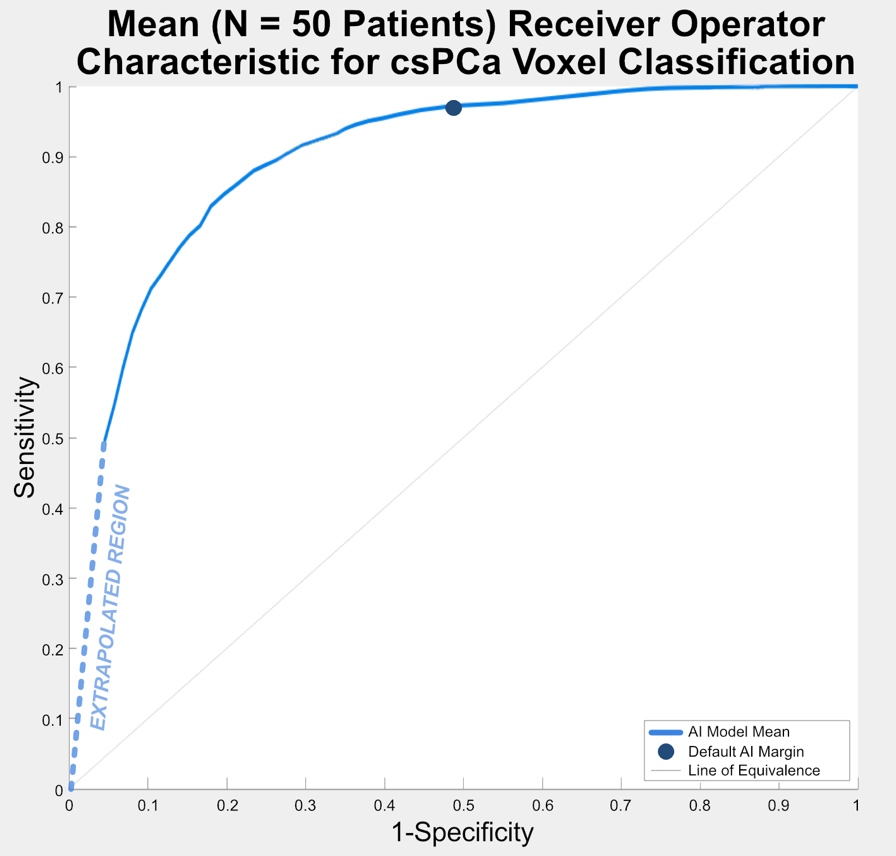


*Supplementary Figure 3: Mean receiver operator characteristic of the artificial intelligence (AI) model for clinically significant prostate cancer (csPCa) voxel classification, computed for each cancer estimation map (CEM) threshold and averaged across the N=50 cases of the independent test dataset.*
